# Molecular epidemiology and transmission dynamics of multi-drug resistant tuberculosis strains using whole genome sequencing in the Amhara region, Ethiopia

**DOI:** 10.1186/s12864-023-09502-2

**Published:** 2023-07-17

**Authors:** Agumas Shibabaw, Baye Gelaw, Mostafa Ghanem, Noah Legall, Angie M. Schooley, Marty K. Soehnlen, Liliana C.M. Salvador, Wondwossen Gebreyes, Shu-Hua Wang, Belay Tessema

**Affiliations:** 1 Department of Medical Laboratory Sciences, College of Medicine and Health Sciences, Wollo University, Dessie, Ethiopia; 2 Global One Health Initiative (GOHi), The Ohio State University, Columbus, OH USA; 3 Department of Medical Microbiology, School of Medical Laboratory Sciences, College of Medicine and Health Sciences, University of Gondar, Gondar, Ethiopia; 4 Michigan Department of Health and Human Services, Infectious disease, Lansing, MI USA; 5 Department of Veterinary Preventive Medicine, College of Veterinary Medicine, The Ohio State University, Columbus, OH USA; 6 Department of Veterinary Medicine, Virginia-Maryland College of Veterinary Medicine, University of Maryland, College Park, MD USA; 7 Institute of Bioinformatics, University of Georgia, Athens, GA USA; 8 School of Animal and Comparative Biomedical Sciences, College of Agriculture and life sciences, University of Arizona, Tucson, AZ USA; 9 Department of Internal Medicine, Division of Infectious Diseases, College of Medicine, The Ohio State University, Columbus, OH USA

**Keywords:** MDR-TB, Genetic diversity, Transmission dynamics, Drug resistance, Molecular epidemiology, Whole genome sequencing

## Abstract

**Background:**

Drug resistant *Mycobacterium tuberculosis* prevention and care is a major challenge in Ethiopia. The World health organization has designated Ethiopia as one of the 30 high burden multi-drug resistant tuberculosis (MDR-TB) countries. There is limited information regarding genetic diversity and transmission dynamics of MDR-TB in Ethiopia.

**Objective:**

To investigate the molecular epidemiology and transmission dynamics of MDR-TB strains using whole genome sequence (WGS) in the Amhara region.

**Methods:**

Forty-five MDR-TB clinical isolates from Amhara region were collected between 2016 and 2018, and characterized using WGS and 24-loci Mycobacterium Interspersed Repetitive Units Variable Number of Tandem Repeats (MIRU-VNTR) typing. Clusters were defined based on the maximum distance of 12 single nucleotide polymorphisms (SNPs) or alleles as the upper threshold of genomic relatedness. Five or less SNPs or alleles distance or identical 24-loci VNTR typing is denoted as surrogate marker for recent transmission.

**Results:**

Forty-one of the 45 isolates were analyzed by WGS and 44% (18/41) of the isolates were distributed into 4 clusters. Of the 41 MDR-TB isolates, 58.5% were classified as lineage 4, 36.5% lineage 3 and 5% lineage 1. Overall, TUR genotype (54%) was the predominant in MDR-TB strains. 41% (17/41) of the isolates were clustered into four WGS groups and the remaining isolates were unique strains. The predominant cluster (Cluster 1) was composed of nine isolates belonging to lineage 4 and of these, four isolates were in the recent transmission links.

**Conclusions:**

Majority of MDR-TB strain cluster and predominance of TUR lineage in the Amhara region give rise to concerns for possible ongoing transmission. Efforts to strengthen TB laboratory to advance diagnosis, intensified active case finding, and expanded contact tracing activities are needed in order to improve rapid diagnosis and initiate early treatment. This would lead to the interruption of the transmission chain and stop the spread of MDR-TB in the Amhara region.

**Supplementary Information:**

The online version contains supplementary material available at 10.1186/s12864-023-09502-2.

## Background

Emerging drug-resistant (DR) *Mycobacterium tuberculosis* threatens tuberculosis (TB) prevention and care efforts globally [1]. Multi-drug resistant (MDR)-TB is defined as resistance to both isoniazid and rifampicin, and rifampicin resistance (RR) requires similar management as MDR-TB [2]. The new World Health Organization (WHO) definition for pre-extensively drug resistance (pre-XDR)-TB is defined as MDR/RR-TB plus resistance to any fluoroquinolones and extensively drug-resistance (XDR)-TB is defined as MDR/RR-TB plus resistance to both fluoroquinolones and either bedaquiline or linezolid [3].

Patients with undiagnosed DR-TB have higher morbidity and mortality than patients with drug-susceptible (DS)-TB disease. Without proper diagnosis and treatment, similar to DS-TB, DR-TB may continue to spread in their communities [4]. Ethiopia is one of the 30 high burden MDR/RR-TB countries globally and ranks 5th among the African countries after South Africa, Nigeria, Mozambique and Democratic Republic of Congo [2]. Management of MDR/RR-TB in Ethiopia is complicated in many areas due to the lack of rapid diagnostic methods drug susceptibility testing (DST) and access to second line drugs (SLDs) for MDR/RR-TB.

It is known that the proportion of MDR-TB is higher among patients who received previous treatment for TB compared to those who are TB treatment-naïve. This disparity is assumed to be due to large proportions of MDR-TB arises from selection of drug resistant mutations during previous ineffective anti-TB treatment period, in contrast to new transmission of pre-existing MDR-TB strains [5–7]. Over time, however, a shift from acquisition of drug resistance during treatment to direct person-to-person transmission of MDR-TB strains occurs [8, 9]. A meta-analysis study on transmissibility of DR-TB compared to DS-TB found that DR-TB results in fewer infections or cases in contacts than DS-TB [10]. It is important to know the transmission dynamics in order to target public health and improve TB prevention and care for the region.

The genome size of *M. tuberculosis* is 4.4 Mbp with low mutation rate and no evidence of horizontal gene transfer [11]. The *M. tuberculosis* complex comprises of seven lineages that vary in geographical distribution around the globe; the strain-types may be specifically adapted to people of different genetic backgrounds [11, 12]. Four lineages are predominant in humans: lineage 1, Indo-Oceanic; lineage 2, East Asian (Beijing spoligotype families); lineage 3, Central Asian Strain (CAS/Delhi spoligotype families); and lineage 4, Euro-American (Latin American–Mediterranean (LAM), Haarlem and the ‘ill-defined’ T spoligotype families) [11]. In addition to lineage 5 and 6, a new *M. tuberculosis* complex lineage, lineage 7 (Aethiops vetus), has identified among strains originated from Ethiopia [13]. Knowing the dominant lineages for particular region can be important to TB prevention and care since the strain type may play a role in disease outcome, variation in vaccine efficacy [14], development of drug resistance [15] and disease epidemiology as it is associated with the presence or absence of clustering due to recent transmission [16].

Classical genotyping tools such as spoligotyping and mycobacterial interspersed repetitive units variable number of tandem repeats (MIRU-VNTR), used alone, can lack sufficient discriminatory power due to high similarity of strains in particular lineages [17, 18]. Spoligotyping method alone is unable to analyze transmission cluster [19, 20]. MIRU-VNTR typing can be a powerful tool to discriminate *M. tuberculosis* lineages and estimation of transmission dynamics [21].

Whole genome sequencing (WGS) has the highest resolution in investigating the transmission chain, with detailed view of genetic diversity as well as determining drug resistant-conferring gene mutations. However, the implementation of WGS is limited especially in high burden countries, due to lack of international standard, shared approaches in universal software for analysis and storage database, as well as financial constraints [22].

The use of WGS gene-by-gene MLST (multi-locus sequence typing) methods and Ridom SeqSphere software has resulted in a more standardized and user-friendly approach than traditional WGS single nucleotide polymorphism (SNP) mapping for resolving and understanding outbreaks [23, 24].

The genotype cluster rate of *M. tuberculosis* strains has been used to assess the efficacy of TB prevention and care programs [25], since clustering is generally a marker for ongoing or recent transmission while unique patterns indicate reactivation of TB disease [25, 26]. In Ethiopia, classical genotyping methods performed on DS-TB have shown complex genetic diversity of *M. tuberculosis* strains [27]. Only a few studies on MDR-TB strain genotypes have been reported with investigations limited by sample size [28] or use of only spoligotyping method [19, 20]. Our finding will help to strengthen TB patients care and management, and revealed the need for improving contact tracing and TB prevention and control strategies in the country. To the best of our knowledge, this is the first in-depth study investigating the molecular epidemiology and transmission dynamics of MDR-TB strains using WGS and MIRU-VNTR in the Amhara region, Ethiopia.

## Materials and methods

### Study setting, design and period

A cohort study was conducted from January 2016 to September 2018 at nine MDR/RR-TB treatment centers in the Amhara region, Ethiopia. A total of 200 patients diagnosed with MDR/RR-TB and treatment failed pulmonary TB patients [29] were enrolled. All the study participants were ≥ 15 years of age. A single sputum sample was collected prior to commencing second-line drug TB treatment. A structured questionnaire was used to collect the following information; MDR/RR-TB contact history, previous history of anti-TB treatment, geographical location (region, zone, district and kebele) and other demographic characteristics of patients. A samples of Lowenstein-Jensen (LJ) culture positive isolates from 45/200 MDR/RR-TB (n = 38) and treatment failed (n = 3) patients were selected for MIRU-VNTR and WGS analyses due to limited funding. The selection criteria were based on the patient’s admission/ data collection year, molecular drug resistance patterns, MDR/RR-TB treatment center hospitals, and geographical location of patients.

### HIV testing

HIV test was done for each study participants using rapid HIV test algorithm of the Federal Ministry of Health of Ethiopia (KHB/STAT-PAK®/Unigold™ or Wantai/Uni-Gold/Vikia) based on the manufacturer instructions. The test was done using finger stick blood sample at the Provider Initiative Counseling and Testing (PICT) services at the respective health institutions in the Amhara region.

### Specimen processing, culture and drug susceptibility test

Sputum collection, transportation, *M. tuberculosis* isolation, identification and DST were performed as previously described [29]. The mycobacteria bacilli DNA was extracted from culture colonies using GenoLyse® DNA extraction kit (Hain Lifescience GmbH, Nehren, Germany). Molecular DST was performed using GenoType® MTBDRplus version 2 kit strips (to detect rifampicin and isoniazid resistance) and GenoType® MTBDRsl version 2 kit strips (to detect fluoroquinolones and aminoglycosides resistance) according to the manufacturer’s instructions (Hain Lifescience GmbH, Nehren, Germany).

### DNA extraction

The genomic DNA of *M. tuberculosis* complex was extracted for MIRU-VNTR and WGS using Cetyl-trimethyl-ammonium Bromide (CTAB) method as described previously [30]. Briefly, sputum samples were inoculated on LJ medium and incubated at 37^o^C until growth becomes clearly visible. One loopful cultured bacterial cells was transferred into the 1.5 ml micro-centrifuge tube containing 400 𝜇L TE (Tris-EDTA) buffer and subjected for heating at 95^o^C water bath for 60 min to kill the cells and then cooled at room temperature. 50 𝜇L lysozyme (10 mg/ml) was then added and incubated at 37^o^C overnight. 70 𝜇L of 10% SDS (Sigma, USA) and 5 𝜇L proteinase K (10 mg/ml; Sigma, USA) were added to the thawed sample and incubated for 10 min at 65^o^C with intermittent mixing. This is followed by the addition of 100 𝜇L of 5 M NaCl and mixed, then 100 𝜇L cetyl-trimethyl-ammonium bromide (CTAB/NaCL) (pre-warmed to 65^o^C in water bath) was added and vortex mixed until the solution becomes milky and then incubated at 65^o^C in water bath for 10 min. Then, 700 𝜇L of chloroform/isoamyl alcohol was added to each tube and the mixture was centrifuged at 13,000 x g for 5 min. The upper aqueous phase of the preparation was transferred into a clean 1.5 ml tube containing 700 𝜇L of cold (-20^o^C) isopropanol and incubated at -20^o^C for 1 h. The solution was centrifuged at 14,000 xg for 20 min and the supernatant was discarded. The tube was washed with 1 ml ice cold (-20^o^C) 70% ethanol by centrifuging at 14,000 xg for 10 min. The supernatant was discarded and the DNA pellet was air dried before being re-suspended in 50 𝜇L TE buffer and kept at -20^o^C until use. The DNA extraction was done at University of Gondar TB culture laboratory and the DNA purity and concentration was checked using NanoDrop™ spectrophotometer machine. The extracted DNA pellet was shipped to The Ohio State University (Ohio, USA) and Michigan Department of Health and Human Services (MDHHS, Michigan, USA) for MIRU-VNTR and WGS typing.

### Genotyping and molecular analysis

#### MIRU-VNTR analysis

MIRU-VNTR 24-loci was performed as described previously [31]. PCR amplification was performed in a total volume of 20 µl containing 2 µl DNA sample and 18 µl reaction mixtures. All reactions were subjected to GeneAmp 2700 thermo-cycler: 95^o^C for 5 min followed by 35 cycles of 1 min at 94^o^C, 1 min at 59^o^C, 1.5 min at 72^o^C and terminated by 10 min at 72^o^C. Genotyping was performed using multiplex PCR with a Rox-labeled MapMarker 1000 size standard (PE Applied BIosystem) for sizing of the PCR products. The PCR fragments were analyzed using ABI 3500xl capillary electrophoresis analyzer (Applied Biosystems, Foster City, California, USA), and the number of copies of each locus was determined by automated assignment using GeneMaper 4.0 software. The number of repeats present in each locus was determined and the results were presented as a set of digits, MIRU code, which reflects the number of MIRU sequence repeats/ alleles. Locus that did not amplify was repeated using singleplex PCR.

Lineage identification of *M. tuberculosis* complex strains was carried out first by best-match analysis with the reference strain with 0.17 maximum cut of distance, and followed by tree based identification on the MIRU-VNTRplus database (https://www.miru-vntrplus.org) [32].A dendrogram was generated using the unweighted pair group method with arithmetic average (UPGMA) and phylogenetic tree analysis was done using MIRU-VNTR typing database. A cluster was defined as two or more *M. tuberculosis* complex isolates sharing identical 24-loci MIRU-VNTR patterns. The recent transmission index was calculated as the number of clustered patients minus number of clusters divided by total number of patients [33]. The discriminatory power was determined by the Hunter-Gaston discriminatory index (HGDI) [34] calculated online using the discriminatory power calculator available at http://insilico.ehu.es. The allelic diversity of each 24-loci MIRU-VNTR was determined in the MIRU-VNTRplus database and grouped into highly (HGDI > 0.6), moderately (0.6 < HGDI > 0.3) and poorly (HGDI < 0.3) discriminatory.

#### WGS analysis

The WGS was performed using NextSeq 550 desktop sequencer (Illumina, San Diego, CA, USA) at MDHHS. Isolated genomic DNA of individual strains was prepared for sequencing using Illumina Nextera XT library preparation kits according to the manufacturer’s instruction (Illumina, San Diego, CA, USA).

#### SNP analysis

Each isolate was sequenced using paired-end sequencing with 4 replicates for each fastq read file, resulting in two sequencing read files (designated by R1 and R2), with 4 replicate each designated by L001, L002, L003 and L004. The final R1 and R2 fastq files of each isolate were created by concatenating their respective 4 sequencing replicates. Variant SNP (vSNP) calling of the *M. tuberculosis* samples was performed using the open source software tool vSNP (https://github.com/USDA-VS/vSNP). The variant SNP pipeline takes as input the two raw reads (R1 and R2) and aligns them to the *M. tuberculosis* H37Rv reference genome (GenBank accession number: NC_000962.3) using Burrows Wheeler Alignment (BWA) [35] and Genome Analysis Toolkit 2.5.2 (GATK) [36–38]. The tool Picard Mark Duplicates was used to remove mapped reads that are redundant and that could potentially lead to erroneous SNP calling (http://broadinstitute.github.io/picard/). The Variant Call Format (VCF) files with the detected SNPs for each isolate was created [39, 40], and VCF filter kept isolates with a Phred quality score above 20 (https://github.com/vcflib/vcflib#vcflib). Proline-glutamate (PE) and proline‐proline‐glutamate (PPE)‐ polymorphic GC‐repetitive sequences (PGRS) were filtered from the analysis [39], as well as SNP positions that had missing data ‘N’, gaps or ambiguous nucleotides. Integrated Genomics Viewer (IGV) [41] provided a way to visually validate the detected SNP sites, and samples that showed no variation in IGV were excluded from the analysis. Maximum likelihood phylogenetic trees were calculated after the curation of SNP sites using RAxML software [42].

#### Core genome MLST (cgMLST) analysis

Raw reads of each samples were aligned to *M. tuberculosis* reference genome H37Rv (RefSeq ID: NC_000962.3) using Burrows Wheeler Alignment. Assembled genomes were uploaded onto the Ridom SeqSphere software version 6.0.2 (Ridom; Münster, Germany). Each isolate sequence was aligned to the Ridom SeqSphere *M. tuberculosis* cgMLST scheme, previously defined consisting of 2891 core genes (GenBank accession number NC_000962.3), for alignment and subsequent genomic analysis [24, 43]. Successful alignments to the cgMLST were defined as good targets by the Ridom SeqSphere software, and full cgMLST analysis was carried out on isolate sequences that conferred > 98% good targets. The cgMLST scheme was also used to compare the 41 *M. tuberculosis* isolates. The resulting phylogeny comparison was made using a Neighbor Joining tree by the Ridom SeqSphere. Minimum spanning tree was created using the same software. The reference genome of *M. tuberculosis* H37Rv (NC_000962.3) was used to root the tree.

#### Genomic relatedness cluster analysis

Pairwise SNP distances were calculated between all sequences using the hamming distance metric, which calculates the number of elements that are dissimilar between two sequences [44]. Genotype clusters were inferred based on how genetically similar two isolates were from each other. The upper thresholds of genomic relatedness or cluster is defined as 12 SNPs or alleles cut off or less and a recent transmission event is defined as 5 or less SNPs or alleles [43, 45]. If two isolates exhibited a distance of more than 12 SNPs or alleles, they were called unique strains.

#### Drug resistance gene mutations analysis

Antimicrobial resistance prediction genetic markers are listed in the TB Profiler database (http://tbdr.lshtm.ac.uk) such as *rpo*B, *rpo*C, *ahp*C, *emb*A, *emb*B, *emb*C, *eth*A, *gid*B, *gyr*A, *gyr*B, *inh*A, *kat*G, *pnc*A, *rps*L, and *rrs* genes [46]. We used the phylo-resistance-search-engine (PhyReSE: http://phyresse.org) and TBprofiler (http://tbdr.lshtm.ac.uk) software to analyze the mutations involved in drug resistance and lineages determination [46, 47].

### Statistical analysis

Data were entered using Epi-Data version 3.1 and exported to SPSS version 20 (SPSS Inc., Chicago, Illinois, USA) for analysis. Data completeness and consistency was checked by running frequencies of each variable. Descriptive statistics and cluster analysis were done.

## Results

### Demographic and clinical characteristics of patients

Among the 45 study participants, the median age was 29 years (IQR: 25–37) and 73% were between 15 and 34 years of age, 60% were male and 20% were HIV-positive. Most of the study participants (67%) had previous history of anti-TB treatment, the literacy rate was 64% and 51% were urban dwellers. In addition, 18% had history of contact with MDR/RR-TB patients, 18% had a family history of TB and 91% were smear positive at the time of diagnosis. Geographically, 42% of MDR-TB patients were recruited from the University of Gondar Referral Hospital and 40% of MDR-TB patients were living in North Gondar zone (Table 1).

**Table 1:** Demographic and clinical characteristics of MDR/RR-TB patients in the Amhara region

Sex	
Male	27 (60)
Female	18 (40)
Age group, years	
15–24	10 (22.2)
25–34	23 (51.1)
35–44	5 (11.1)
45–54	5 (11.1)
>55	2 (4.4)
Residence	
Urban	23 (51)
Rural	22 (49)
Previous ant-tuberculosis treatment	
Yes	30 (66.7)
No	15 (33.3)
Previous contact with MDR/RR-TB patients	
Yes	8 (17.8)
No	37 (82.2)
Family history of tuberculosis	
Yes	8 (17.8)
No	37 (82.2)
HIV status	
Positive	9 (20)
Negative	36 (80)
Educational status	
Literate	29 (64.4)
Illiterate	16 (35.6)
MDR/RR-TB treatment center hospitals	
University of Gondar Hospital	19 (42.2)
Boru Meda Hospital	6 (13.3)
Woldia Hospital	6 (13.3)
Ataye District Hospital	5 (11.1)
Finote Selam Hospital	2 (4.4)
Metemma Hospital	3 (6.7)
Debre Bbirhan Hospital	2 (4.4)
Debre Tabor Hospital	1 (2.2)
Debre Markos Hospital	1 (2.2)
Zonal location in the Amhara region	
North Gondar	18 (40)
South Gondar	1 (2.2)
East Gojjam	1 (2.2)
West Gojjam	6 (13.3)
North Wollo	6 (13.3)
South Wollo	6 (13.3)
North Shewa	5 (11.1)
Oromia Kemissie Special	2 (4.4)
Years of patient participation (labelling)^**^	
2016	3 (6.7)
2017	9 (20)
2019	33 (73.3)

^**^ Labelling of patient’s participated and isolates identified during data collection period in years are as follow: Patients participated and isolates identified in 2016 (A19, A34 and A141 isolates); 2017 (A29, A66, A73, A74, A76, A126, A132, A143 and A144 isolates); 2018 (A85, A88, A90, A92, A99, A101, A104, A106, A110, A113, A114, A115, A116, A117, A122, A158, A161, A162, A166, A167, A170, A171, A172, A173, A175, A177, A180, A184, A188, A189, A191, A192 and A194 isolates)

### Population structure of *M. tuberculosis*

#### Analysis by MIRU-VNTR

A total of 45 MDR/RR-TB isolates were submitted for MIRU-VNTR 24-loci testing. Two isolates were excluded from the final MIRU-VNTR analysis. One isolate did not have a PCR amplicon for more than 4 loci and the other isolate was contaminated and was not identified as *M. tuberculosis*. Isolates with no PCR amplicon at only one locus were included into the analysis and categorized under missing data. Three isolates showed double alleles in a single locus.

**Fig. 1:**
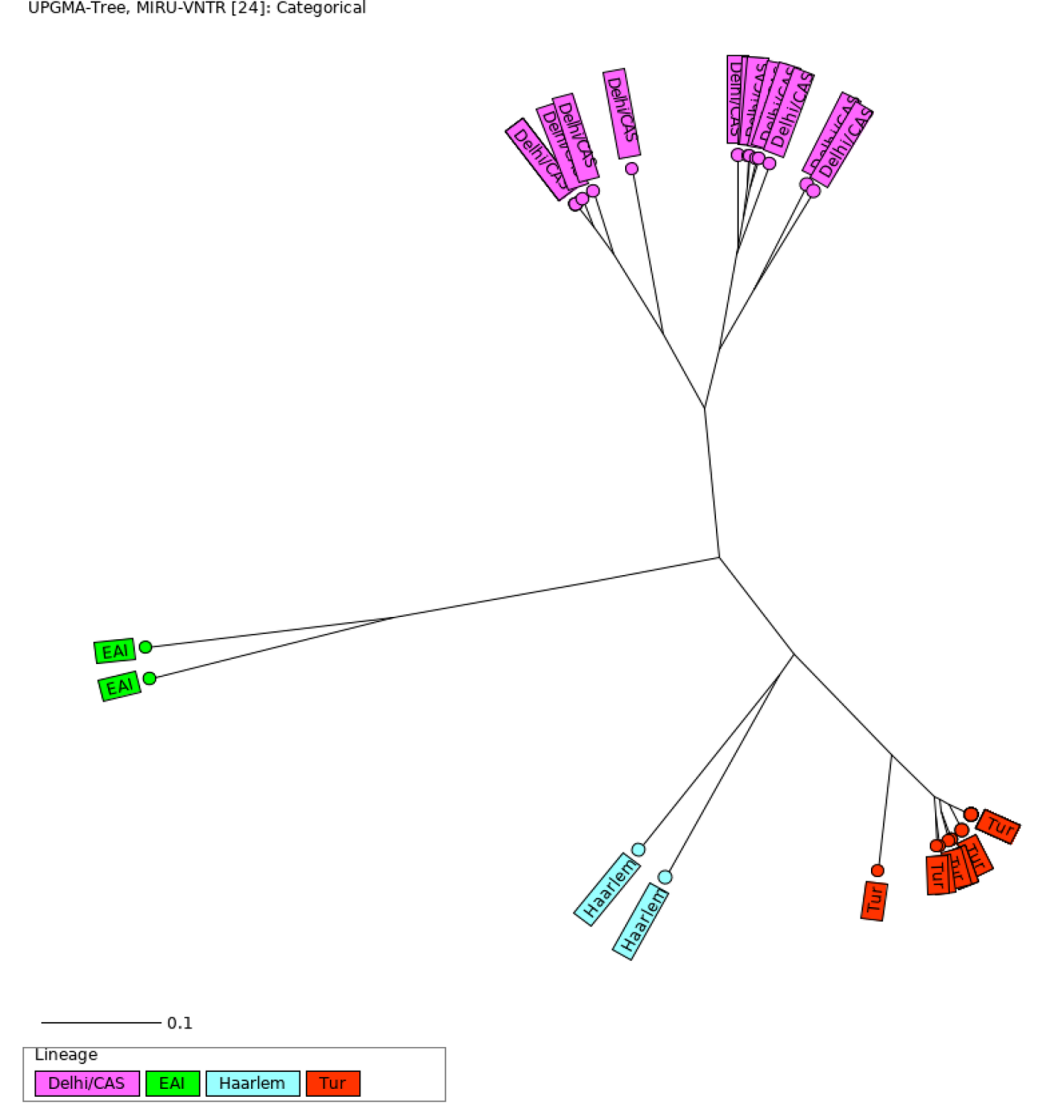
Radial UPGMA tree based on the MIRU-VNTR 24-loci copy numbers. The tree was calculated using MIRU-VNTRplus website. EAI: East African Indian/ Indo-Oceanic

The most prevalent *M. tuberculosis* lineages were TUR (23/43; 53.5%) and Delhi/CAS (16/43; 37.2%). Haarlem and Endo-Oceanic both accounted for 4.6% (2/43) (Fig. 1, Supporting file: Fig S1).

Twenty-four of the 43 (55.8%) *M. tuberculosis* isolates were clustered into four different MIRU-VNTR typing groups containing 2, 3, 5 and 14 strains in each. Nineteen isolates had unique MIRU-VNTR patterns. The recent transmission index using MIRU-VNTR pattern analysis was 46.5%.

Out of the 24 loci, the least (low) allelic diversity (h < 0.3) was seen in 9 loci (MIRU 02, MIRU 04, MIRU 20, Mtub29, ETRB, MIRU 23, MIRU 24, MIRU 27 and Mtub34). Moderate allelic diversity (h = 0.6 − 0.3) was seen in 14 loci (Mtub04, ETRC, MIRU 40, MIRU 10, MIRU 16, Mtub21, QUB-11b, ETRA, MIRU 26, MIRU 31, Mtub39, QUB-26, QUB-4156 and MIRU 39). The highest allelic diversity indices (h > 0.6) was seen only for Mtub30.

**Fig. 2:**
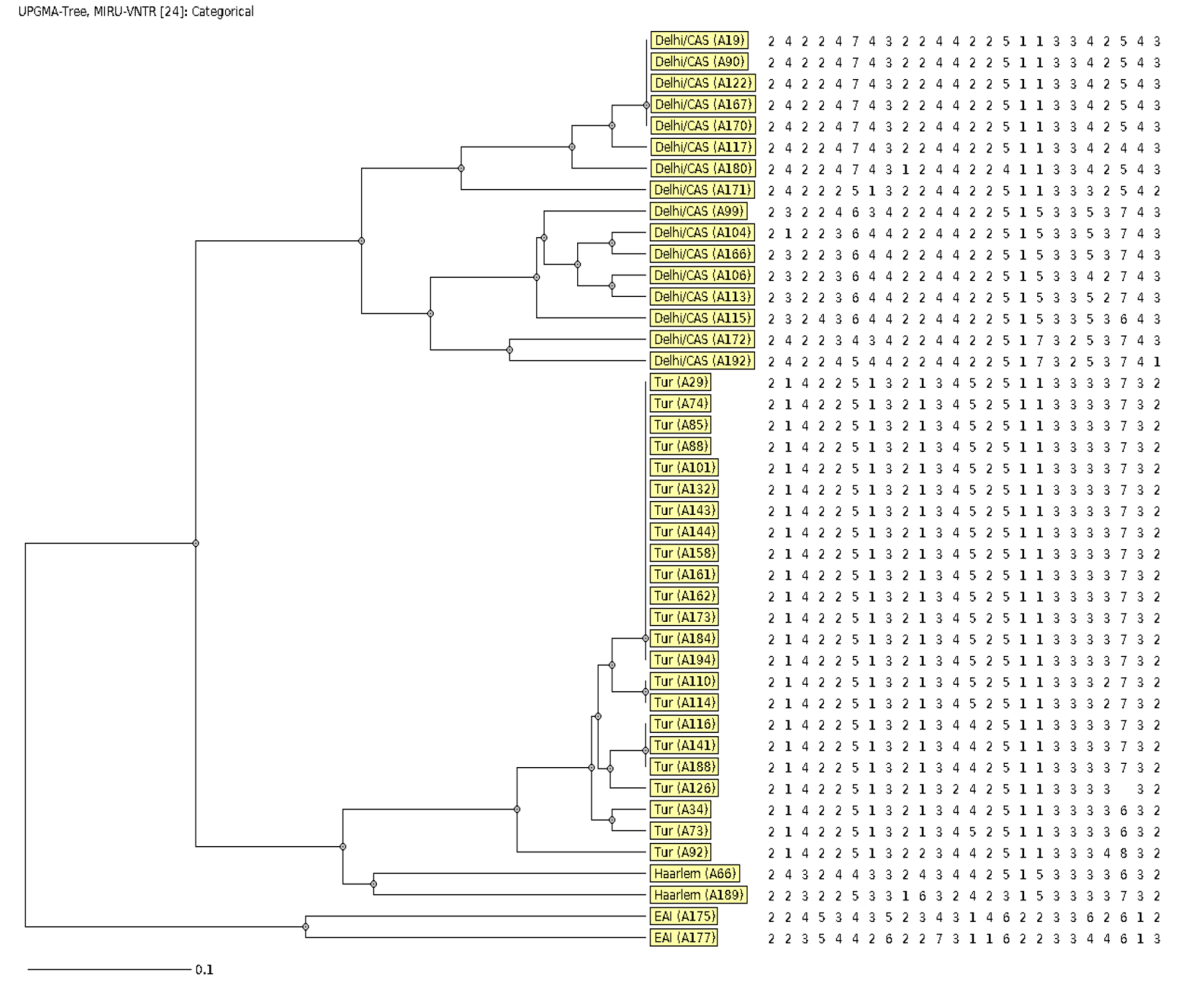
Phylogenetic tree based on 24-loci MIRU-VNTR pattern of 4 clustered MDR/RR-TB isolates. The VNTR loci copy number for each isolates with sub-lineage of *M. tuberculosis*

Phylogenetic relationship with representative branch lengths demonstrates 4 clusters of MDR/RR-TB isolates based on the 24-loci VNTR pattern (Fig. 2).

#### Analysis by WGS

Four of the 45 samples (A126, A132, A143 and A191) had poor coverage and/or contaminated or one isolate was not identified as *M. tuberculosis*, and excluded from the final analysis. Of the 41 remaining isolates, the statistics used to summarize the data showed that the isolates exhibited read-quality scores between 32 and 33 Q, over 99% genome coverage, average sequencing read-length of 142 bp, and an average coverage of 97.1 depth.

**Fig. 3:**
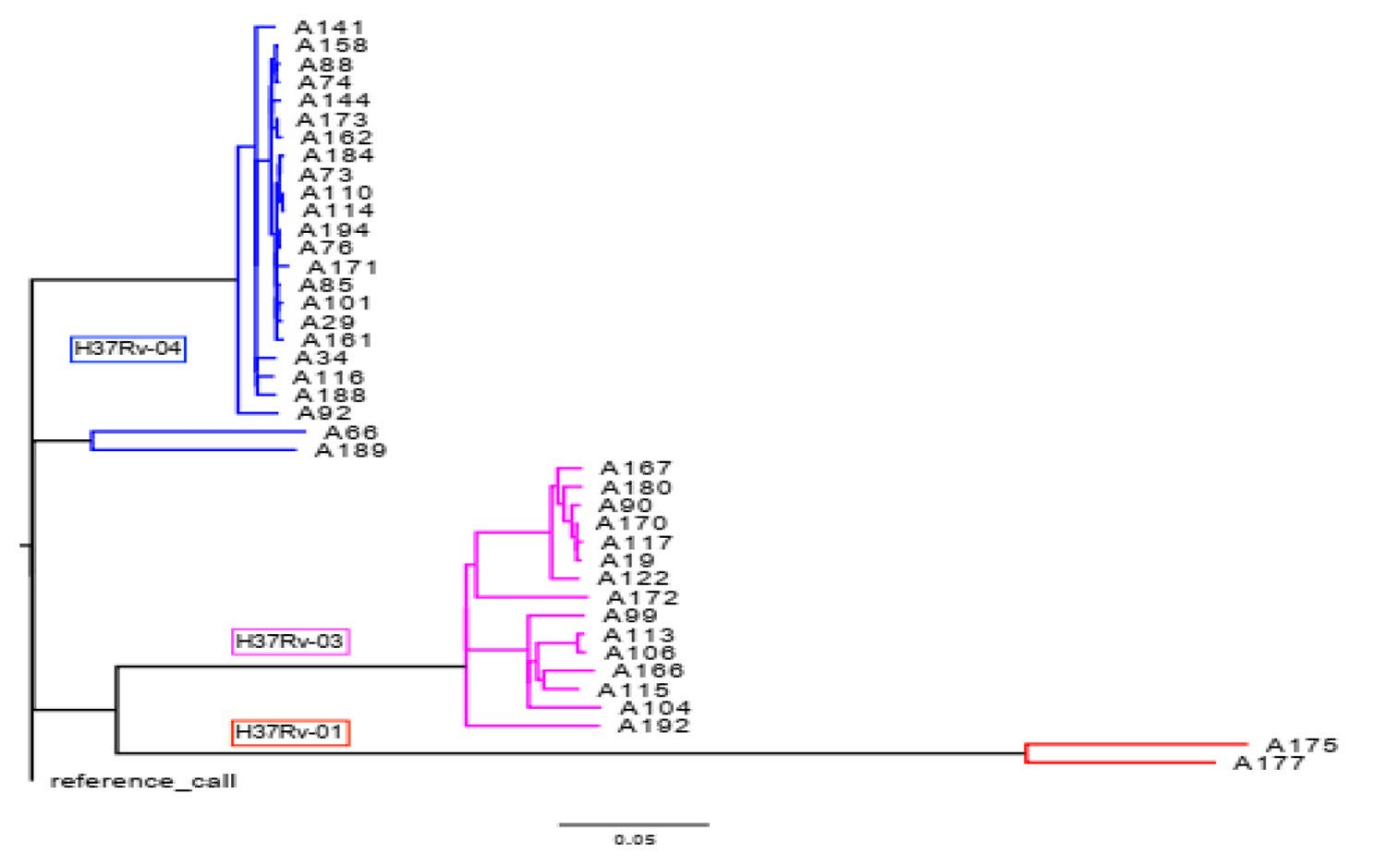
Maximum likelihood of *M. tuberculosis* phylogeny. Three clades were identified and colored to represent the *M. tuberculosis* lineages: H37Rv-04/lineage 4 (n = 24, blue branches), H37Rv-03/lineage 3 (n = 15, magenta branches), and H37Rv-01/lineage 1 (n = 2, red branches)

A total of 4456 SNP sites were identified. The maximum likelihood tree identified 3 distinct clades based on *M. tuberculosis* lineage classification in accordance with bioinformatics pipeline vSNP or SNP based barcode nomenclature [11] (Fig. 3). Overall, three major *M. tuberculosis* lineages were identified; the predominant lineage was Euro-American (lineage 4) accounted for 58.5% (24/41), followed by 36.6% (15/41) lineage 3 (Delhi/CAS) and 5% (2/41) Endo-Oceanic lineage (lineage 1).

The predominant sub-lineages of Euro-American lineage was TUR, 92% (22/24) (Coll sub-lineage 4.2.2). Haarlem (Coll sub-lineage 4.1.2) and X-type (Coll sub-lineage 4.1.1) each accounted 4% (1/24). Isolates belonging to lineage 3 harbored the CAS specific RD750 deletion and its sub-lineage was identified as CAS for 53% (8/15) and CAS1-kili for 47% (7/15) spoligotypes. Lineage 1 isolates had RD239 deletion and contain two spoligotype families (EAI3 and EAI5).

**Fig. 4:**
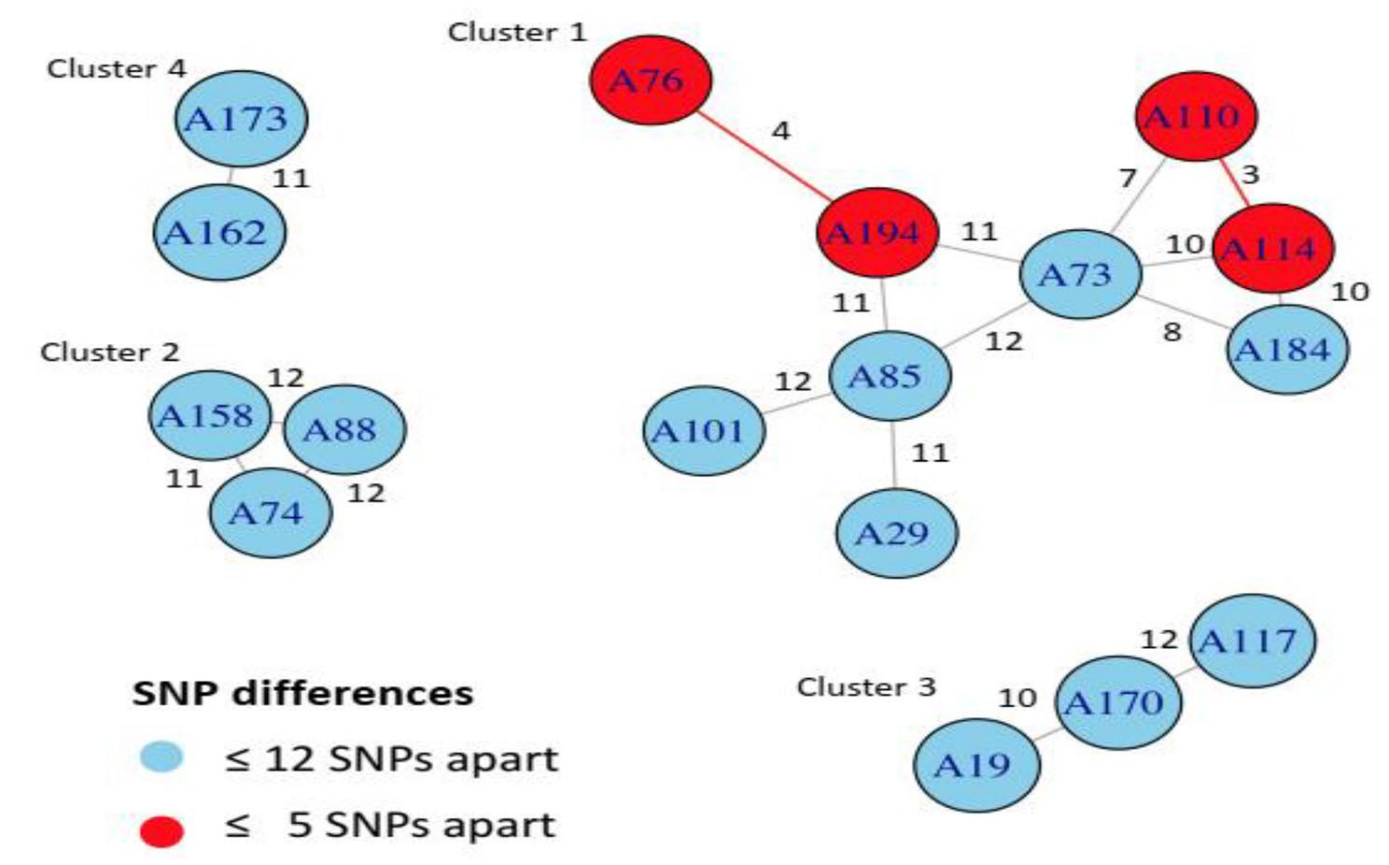
Cluster analysis based on pairwise SNP difference between clustered isolates. The number indicates the SNP differences that exist between two isolates. Red circle indicates recent transmission links between isolates and the blue circle shows isolates within a transmission cluster

41% (17/41) of the isolates were clustered into four WGS groups (Fig. 4). The remaining isolates were more than 12 SNPs apart, known as unique strains. Compared to the lineages identified by vSNP, cluster 1 is composed by nine isolates belonging to lineage 4 with two recent transmission links (A76-A194 and A110-A114) denoted as red circles in Fig. 4. Clusters 2 and cluster 3 has 3 isolates each belonging to lineages 4 and 3, respectively. Cluster 4 is composed of two isolates belonging to lineage 4.

**Fig. 5:**
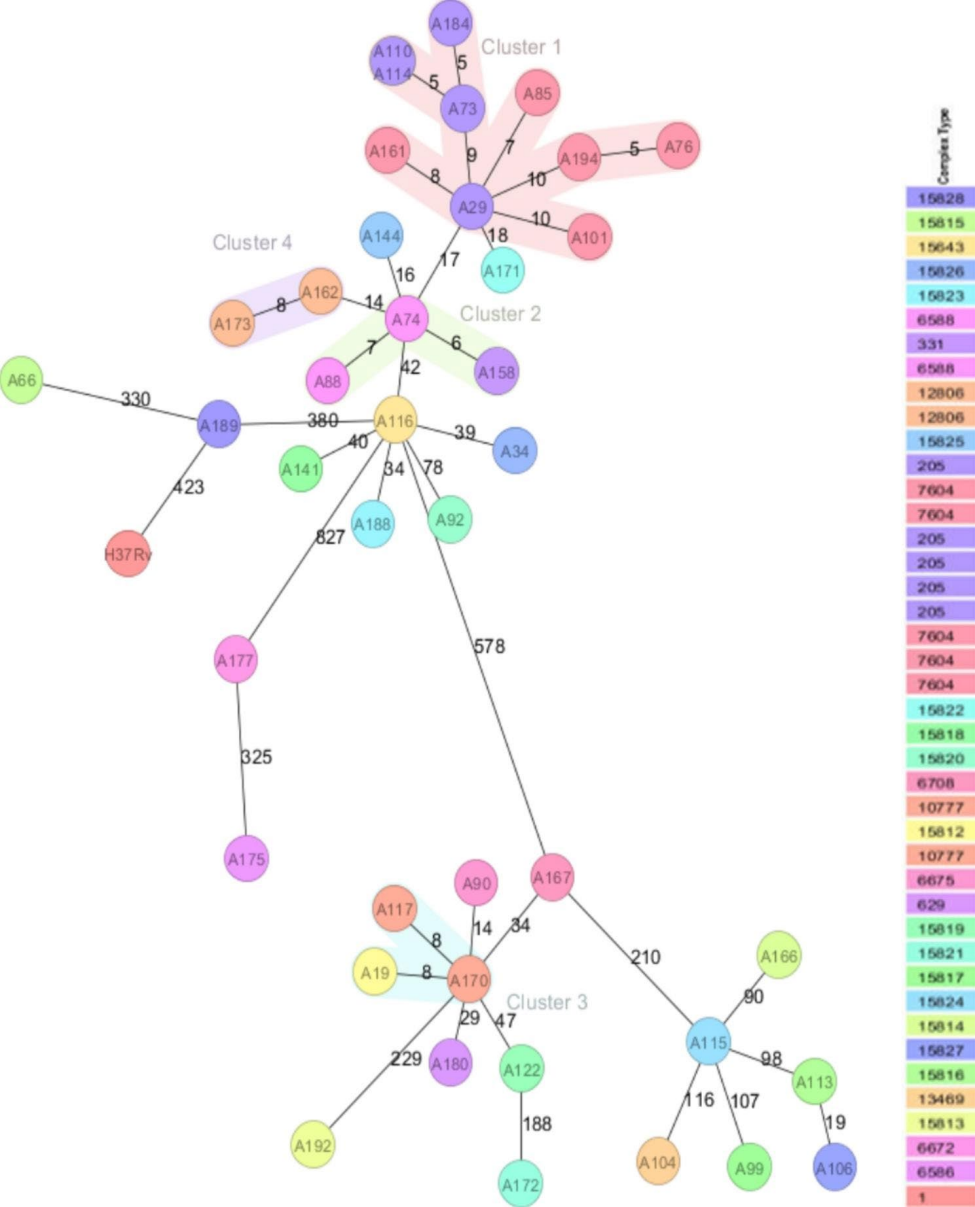
Minimum spanning tree for 41 *M. tuberculosis* isolates based upon cgMLST analysis. *M. tuberculosis* cgMLST complex type / cluster-alert distance were 12 alleles. The numbers displayed on lines between circles represent the numbers of different alleles between different samples. Samples were grouped by color according to their cgMLST complex type (right side)

Four different cgMLST clusters were identified within the 41 WGS-genotyped isolates; 18 isolates were clustered and 23 isolates were unique. The upper limit of cluster threshold was defined as a maximum of 12 allele difference (Fig. 5, Supporting file: Fig S2).

Cluster 1 had 10 isolates (A184, A73, A110, A114, A29, A161, A101, A194, A76 and A85); cluster 2 had 3 isolates (A158, A74, and A88); cluster 3 had 3 isolates (A117, A19, and A170) and cluster 4 had 2 isolates (A173 and A162). Two recent transmission events were identified in cluster 1 (A76-A194, and A110-A114-A73-A184).

In total, 4 WGS transmission clusters in both SNP and cgMLST based analysis corresponding to a clustering proportion of 44% (18/41) and resulting in a clustering rate or recent transmission index of 34% (calculated as (18 − 4)/41)).

45% of TUR lineage was resistance to all first line drugs and 73% had previous history of anti-TB treatment (Table 2).

**Table 2:** *M. tuberculosis* lineages compared to anti-TB drug resistance, patients’ previous contact to MDR/RR-TB, previous anti-TB treatment and HIV status

*M. tuberculosis* lineages (N)	Anti-TB drug resistance									PT n (%)	MDR/RR-TB contact n (%)	HIV + n (%)
	RIF n %	INH n %	PZA n %	EMB n %	STR n %	FLDR n %	ETH n %	FQs n %	SLI n %			
TUR (22)	21 (95)	21 (95)	14 (64)	19 (86)	15 (68)	10 (45)	17 (77)	0	1 (5)	16 (73)	6 (23)	2 (9)
Delhi/CAS (15)	13 (87)	13 (87)	4 (27)	10 (67)	7 (47)	4 (27)	5 (33)	1 (7)	0	11 (73)	1 (7)	4 (27)
EAI (2)	2 (100)	2 (100)	2 (100)	2 (100)	2 (100)	2 (100)	1 (50)	0	2 (100)	1 (50)	0	2 (100)
Haarlem (1)	1 (100)	1 (100)	0	0	1 (100)	0	1 (100)	0	0	1 (100)	0	0
X-type (1)	1 (100)	1 (100)	0	1 (50)	1 (100)	0	1 (100)	0	0	1 (100)	0	1 (50)

EAI: Endo-oceanic lineage, PT-previous anti-TB treatment, RIF- rifampicin, INH- Isoniazid, PZA- pyrazinamide, EMB- Ethambutol, STR-Streptomycin, FLDR- Resistance to all first-line drugs, ETH-Ethionamide, FQs-Fluoroquinolones, SLI- Second-line injectable drugs, + =positive for HIV

## Discussion

We analyzed 45 MDR/RR-TB isolates collected from 2016 to 2018 from the Amhara region health institutions. However, only 41 isolates were eligible for further analysis and the remaining four isolates were excluded from WGS analysis.

Transmission clusters analysis is the basis for tracing outbreak of *M. tuberculosis* clones and to improve TB control policies and strategies. Clustering is an indicator of recent transmission [48]. Recent transmissions association with MDR-TB strains has been reported in various geographical settings [26, 28, 49–51].

The present study suggests possible high transmission of MDR/RR-TB in the Amhara region, as shown by 44% (18/41) and 55.8% of MDR-TB isolates being clustered based on SNPs/alleles difference and VNTR genotype, respectively. This variation is due to the fact that WGS generates smaller clusters than MIRU-VNTR typing. As a result, MIRU-VNTR typing is less predicative of close genetic relatedness compared with WGS and has led to overestimation of TB transmission in the community [52].

Although an extended contact evaluation or epidemiological link data were not collected to determine direct transmission links, the majority of the patient characteristic within the transmission clusters obtained from SNPs or allele difference matched with variable degree the patients’ admission/ treatment initiation dates within one year apart and/or proximity of location within hospitals and/or zones or Districts.

A recent MDR-TB transmission index can be used to assess the country’s efficacy of TB prevention and care programs. Looking specifically the four recent transmissions grouped isolates using SNPs (A76-A194 and A110-A114), the linked patients live in the same district area (two were in Achefere and the other two were in Tegedie district). It is unknown if the patients may have had direct contact history and/or social interaction with each other but the geographical proximity supports epidemiological transmission links. Interestingly, cgMLST also identified the same two recent transmission clusters (A76-A194 and A110-A114-A73-A184). The first one is same as SNPs analysis but the second has a total of 4 patients compared to two by SNPs. Patients, labelled as A73 and A184, were not living in the same geographic location like patients labelled as A110 and A114, but one of the patient was admitted at the same hospital with others and lives in the same zone but not in the same district like others.

In our finding, the MDR-TB clustering rate (34%) is in concordance with other countries such as China (33.7%) and Saudi-Arabia (32%), indicating high rate of drug resistance TB transmission [53, 54]. Compared to our finding, previous study have reported higher cluster rate of MDR-TB transmission in Ethiopia (86-86.5%), Turkey (79%), Saudi-Arabia (67.6%), Tunisia (65%) and Europe (43%) [19, 20, 26, 49, 51, 55]. MDR-TB cluster rates lower than our studies have also been reported in the US (20%) and in the UK (15%) [56, 57]. The disparity between studies in the transmission cluster rate might be due to differences in genotyping methods, types of TB patients (DS- vs. MDR or XDR-TB), geographical location, variation in TB intensive contact evaluation and social interaction or network and population movement. In our study, the TUR strains are more clustered compared to the other strains.

In Ethiopia, the health extension program is functioning well at the community level that results for early detection and treatment of TB suspected patients within few weeks of onset of TB symptoms. But the relatively higher clustering rate observed in our study might be linked to insufficient TB prevention and care programs in Ethiopia or the region. Given the new genotyping cluster data, a recommendation to the regional TB program to increase active tracing of contacts of patients are needed in conjunction with improving the diagnostic facilities for MDR-TB. Lastly, early treatment of infected patients is essential to block the transmission chain. Amhara region has only two TB culture laboratories. It is very important to expand the capabilities for TB culture and DST to combat the spread of drug resistant TB in Ethiopia and in the Amhara region.

Our finding revealed that Euro-American lineage (lineage 4) was the most dominant among MDR/RR-TB strains in the Amhara region, followed by Delhi/Central Asia (lineage 3) and Endo-oceanic lineage (lineage 1) using WGS analysis. The main lineage classifications are the same in the MIRU-VNTR analysis except one sub-lineage classification variation: One isolate was Haarlem by MIRU-VNTR but X-type by WGS analysis. Our finding also revealed the dominance of lineage 4 that is already explained previously that Euro-American lineage is mainly associated with drug resistance [58].

A recent meta-analysis study of Ethiopia shown that Euro-American lineage was predominant in Ethiopia, followed by lineage 3/Delhi/CAS and lineage 1 in all TB forms [59]. Other studies reported that Euro-American and EAI lineage were predominant among MDR-TB strains in Ethiopia, Euro-American strains accounted 52% and 36% using sploligotyping alone [19, 20] and 83% from 12 strains in Ethiopia [28] and other countries reported, 55%, 54% and 100% in Saudi Arabia, Lebanon and Tunisia, respectively [49, 51, 60]. No Beijing strain was present in our study although it is commonly associated with drug resistance. This is in contrast to studies in Belarus, Turkey and Central Asia, which reported the dominance of Beijing strains and elucidated its association with drug resistance [26, 61, 62]. This might be attributed to that different lineages are prevalent in specific regions/countries and adapted to a particular human populations/ host-pathogen compatibility/ and more likely to transmit and cause disease of the same ethnicity [12].

The most prevalent genotypes within the Euro-American main lineage were sub-lineage TUR (92%; 22/24) and Haarlem (4%) and X-type (4%) strains. From lineage 3, we found CAS (53%) and CAS1-kili (47%) spoligotype families and two EAI3 and EAI5 spoligotype families were found under lineage 1 category. Although Haarlem is one of the widespread sub-lineage globally, the TUR sub-lineage is geographically restricted mainly in Asia and Africa [63]. Previous studies in Ethiopia reported the presence of Haarlem, CAS and EAI strains in MDR-TB isolates characterized by limited sample size or use of spoligotyping technique alone. TUR strains (designated previously as LAM7-TUR) have not been reported yet among MDR-TB strains in Ethiopia. Large cluster of TUR strains have been identified in Turkey and Saudi Arabia [49, 50]. The presence of high percentage of TUR strains in Ethiopia may be explained by the introduction of these strains from Turkey/Saudi Arabia to Ethiopia due to the growing economic partnership between Ethiopia and Turkish and/or Saudi Arabia or due to the return of the Ethiopian immigrants from Saudi Arabia to Ethiopia. Another theory proposed by Comas et al. that proposed an African origin for *M. tuberculosis* complex with some genotypes already present on the African continent before migration to European and Asia [27], and so this lineage could be emerged in Ethiopia.

In addition, two of the pre-XDR-TB strains were lineage 1 and one isolate in each represented the TUR and Delhi/CAS strains. From 3 non-MDR-TB isolates, two of them were grouped in lineage 3 and sub-lineage CAS and the other one isolate was TUR strain. The Pre-XDR-TB strains are grouped under the three lineages. The three unique strains were not found in a cluster that might be due to de novo evolution of resistance. This hypothesis might be in agreement with the hypothesis that XDR-TB acquisition appeared mainly by *de novo* evolution of resistance in addition to patient to-patient transmission [62].

One of the limitations of this study is the lack of extended epidemiological links data between the clustered cases and thus unable to confirm the genotype clusters and small number of MDR-TB isolates were genotyped (due to limited funding) that may not be representative of the region and may affect the cluster analysis. Genotyping of 200 MDR/RR-TB isolates may identify additional clusters. Plan is underway to genotype all of the remaining isolates.

## Conclusions

Our finding showed that Amhara region appears to have ongoing MDR/RR-TB transmission dynamics indicated by high cluster proportion of MDR-TB isolates. Three major lineages were found among MDR-TB isolates in the Amhara region with three dominant sub-lineages. Improved TB infection control with expanded contact investigation, early diagnosis and treatment with appropriate regimen will be crucial to stop the growing spread and interrupt the transmission chain of MDR-TB strains. Improved TB surveillance, diagnosis and treatment of DS-TB will decrease treatment failure and re-infection of community.

## Electronic supplementary material

Below is the link to the electronic supplementary material.

**Supplementary Material**: **Fig S1**: Minimum spinning tree based on the 24-loci MIRU-VNTR typing data of 43 *M. tuberculosis* isolates in the Amhara region, generated by MIRU-VNTRplus website (minimum size: two VNTR types). The size of each circle is proportional to the number of MIRU-VNTR types belonging to a particular complex. Classification of the isolates into the different phylogenetic lineages is visualized by color coding. **Fig S2**: A neighbor joining tree showing relatedness between 41 isolates based on the M. tuberculosis complex cgMLST v2.1; 2891 targets. *M. tuberculosis* H37Rv genome was used to root the tree. Samples were grouped by color according to their cgMLST complex type.

## Data Availability

Raw sequence data are available under the project accession number PRJNA935744 (https://www.ncbi.nlm.nih.gov/sra/PRJNA935744).

## List of abbreviations

TB: Tuberculosis
MDR-TB: Multi-drug resistant tuberculosis
WGS: Whole genome sequence
SNP: Single nucleotide polymorphisms
MIRU-VNTR: Mycobacterial interspersed repetitive unit variable number of tandem repeats
