# Supplementary material for: Molecular epidemiology and transmission dynamics of multi-drug resistant tuberculosis strains using whole genome sequencing in the Amhara region, Ethiopia
Source: BMC Genomics. 2023 Jul 17;24:400. doi: 10.1186/s12864-023-09502-2 (PMC10351181; doi:10.1186/s12864-023-09502-2)
Supplement: Supplementary file 1 — Supplementary Material: Fig S1: Minimum spinning tree based on the 24-loci MIRU-VNTR typing data of 43 M. tuberculosis isolates in the Amhara region, generated by MIRU-VNTRplus website (minimum size: two VNTR types). The size of each circle is proportional to the number of MIRU-VNTR types belonging to a particular complex. Classification of the isolates into the different phylogenetic lineages is visualized by color coding. Fig S2: A neighbor joining tree showing relatedness between 41 isolates based on the M. tuberculosis complex cgMLST v2.1; 2891 targets. M. tuberculosis H37Rv genome was used to root the tree. Samples were grouped by color according to their cgMLST complex type. [file 12864_2023_9502_MOESM1_ESM.doc]

**Supplementary Materials**

**
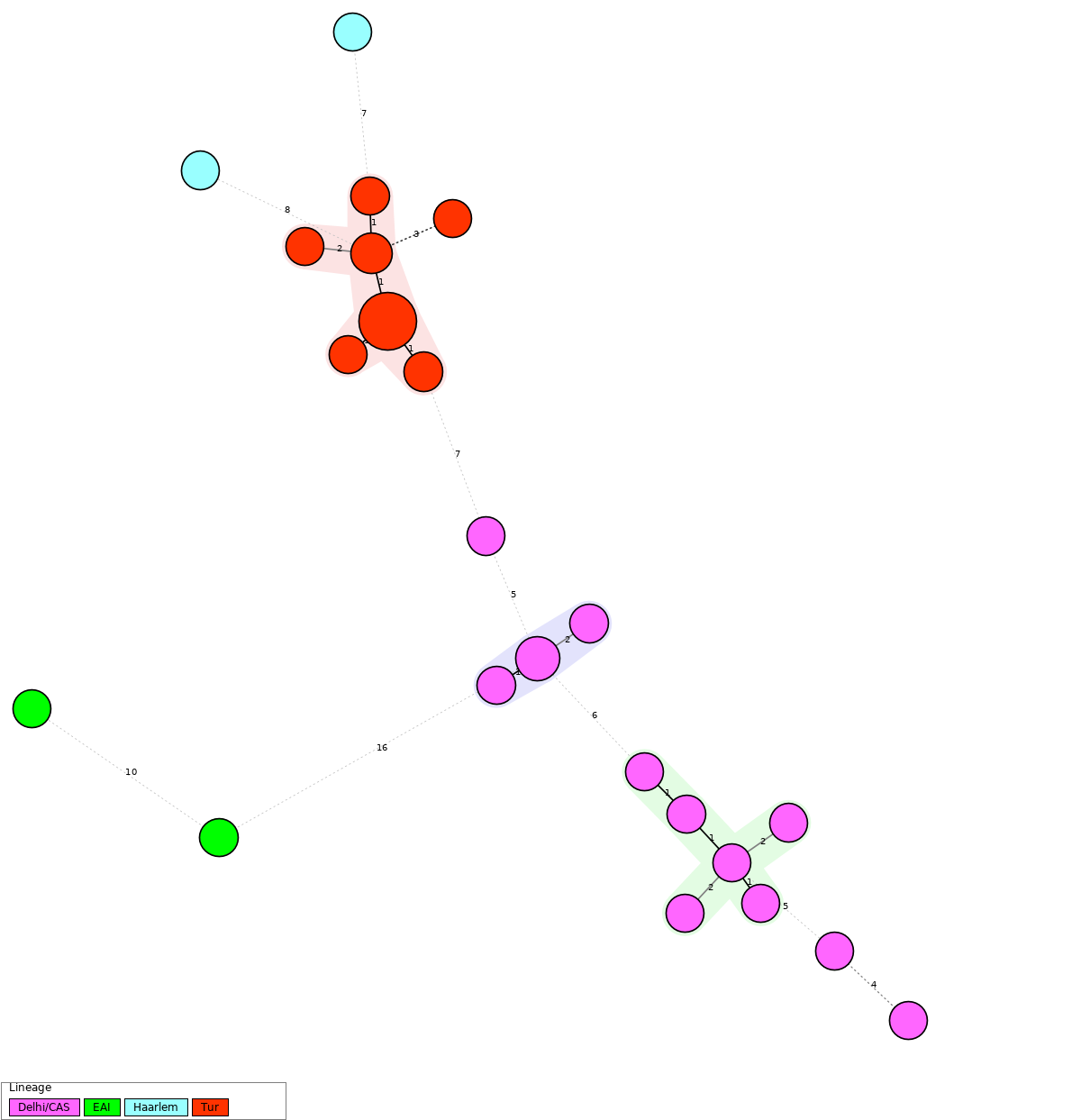
**

**Fig S1:** Minimum spinning tree based on the 24-loci MIRU-VNTR typing data of 43 *M. tuberculosis* isolates in the Amhara region, generated by MIRU-VNTRplus website (minimum size: two VNTR types). The size of each circle is proportional to the number of MIRU-VNTR types belonging to a particular complex. Classification of the isolates into the different phylogenetic lineages is visualized by color coding.


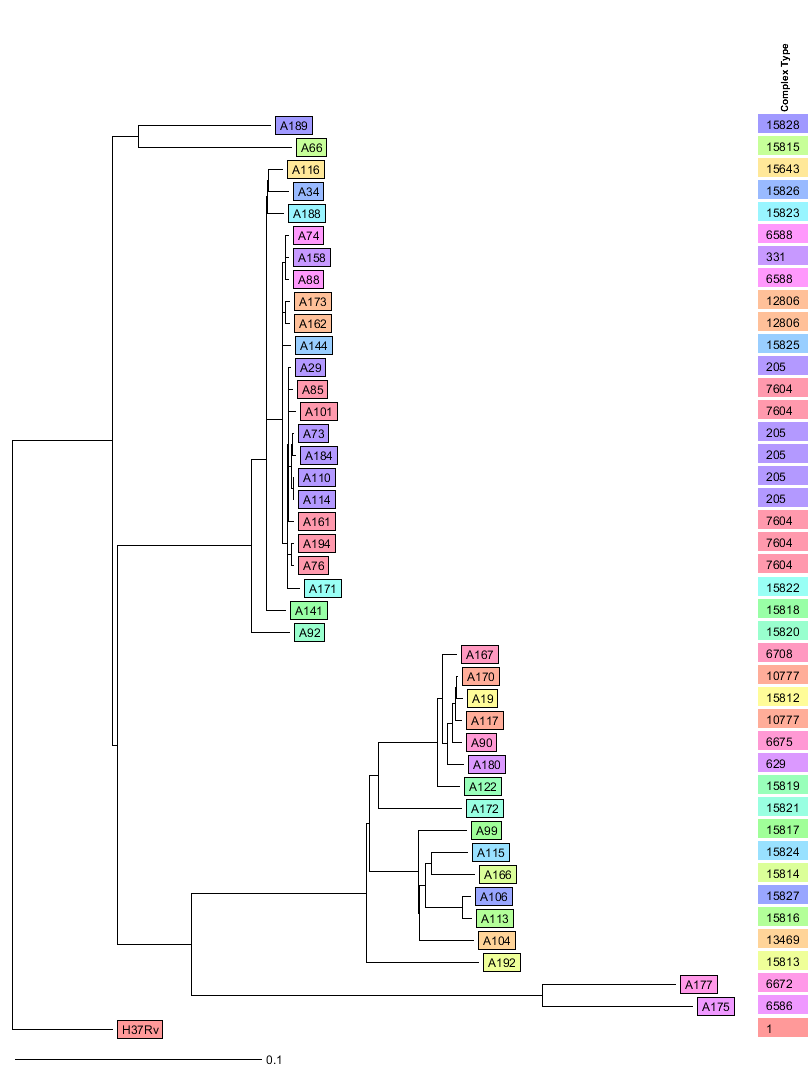


**Fig S2**: A neighbor joining tree showing relatedness between 41 isolates based on the *M. tuberculosis* complex cgMLST v2.1; 2891 targets. *M. tuberculosis* H37Rv genome was used to root the tree. Samples were grouped by color according to their cgMLST complex type.
